# Rare diseases: human genome research is coming home

**DOI:** 10.1101/mcs.a006210

**Published:** 2022-02

**Authors:** Hans-Hilger Ropers, Clara D. van Karnebeek

**Affiliations:** 1 Max Planck Institute for Molecular Genetics, Berlin D-14195, and Institute of Human Genetics, University Medicine, Mainz D-55131, Germany;; 2 Departments of Pediatrics and Human Genetics, Emma Children's Hospital, Amsterdam University Medical Centers, NL-1105 AZ Amsterdam, The Netherlands

## Abstract

After a long and largely disappointing detour, Genome Research has reidentified Rare Diseases as a major opportunity for improving health care and a clue to understanding gene and genome function. In this Special Issue of *CSH Molecular Case Studies* on Rare Diseases, several invited Perspectives, numerous Case Reports, and this Editorial itself address recent breakthroughs as well as unsolved problems in this wide field. These range from exciting prospects for gap-free diagnostic whole-genome sequencing to persisting problems related to identifying and distinguishing pathogenic and benign variants; and from the good news that soon, the United Kingdom will no longer be the only country to have introduced whole-genome sequencing into health care to the sobering conclusion that in many countries the clinical infrastructure for bringing Genome Medicine to the patient is still lacking. With less than 5000 genes firmly implicated in disease, the identification of at least twice as many disease genes is a major challenge, and the elucidation of their function is an even larger task. But given the renewed interest in rare diseases, their importance for health care, and the vast and growing spectrum of concepts and methods for studying them, the future of Human Genome Research is bright.

## THE HUMAN GENOME PROJECT: FROM RARE TO COMMON DISEASES

That genome sequencing would be key to the diagnosis, prevention, and even cure for many of the genetic disorders listed in Victor McKusick's catalog of Mendelian phenotypes (McKusick 2007)^3^ was an important driving force for establishing the Human Genome Project (HGP) in 1990 (McKusick 1982, 1992). About halfway to its completion, however, the orientation of this project changed. Paradoxically, this happened when several dozens of well-known Mendelian disorders, including Duchenne muscular dystrophy, cystic fibrosis, fragile X mental retardation, myotonic dystrophy, and Huntington's chorea, had already been elucidated by positional cloning strategies, maybe partly because of the belief that most low-hanging fruit had already been reaped. More importantly, however, common disorders such as dementia, schizophrenia, diabetes, autism, cardiovascular disease, and hypertension were more appealing and easier to “sell” to the public and policy makers alike, which is why they were soon promoted as the new target of human genome research (Risch and Merikangas 1996; Collins et al. 1998). Although it secured public support and continued funding for the HGP, the scientific underpinning of this move was rather weak, as it was based on the catchy but unproven common disease–common variant (CD/CV) hypothesis—that is, the assumption that for common diseases, common genetic risk factors must exist. And a long-lasting legacy of this decision was that the conceptually sound and successful research into Mendelian disorders remained unpopular and vastly underfunded until well into the second decade of this millennium.

Through the years, billions were spent in search of common genetic variants and on genome-wide association studies (GWASs) to identify diagnostic and predictive markers of common diseases, despite growing concern about the doubtful validity of this concept and the meager yield of these efforts. However, because of the large number of prominent proponents of this research and their overly optimistic predictions (Bell 1998; Chakravarti 1999; Goldstein et al. 2003; Collins 2005), those critical voices were rarely heard. By 2003, several hundred thousand single-nucleotide polymorphisms (SNPs) had been identified (International HapMap Consortium 2003) and 5 years later, GWASs in large cohorts of patients and healthy individuals had indeed revealed significantly disease-associated genetic markers for a whole range of common diseases (Manolio et al. 2008). However, because of their low frequency and/or too low odds ratios, the vast majority of these SNPs turned out to be unsuitable as diagnostic or predictive markers. Also, the hope of the GWAS community that these markers would be a rich source of information about the molecular basis and the pathogenesis of the associated diseases has not materialized, except for a handful of common disorders (Ropers 2007; Maher 2008).

## IT IS TIME TO TURN THE PAGE

In hindsight, there are plausible explanations for this sobering outcome. First, factors such as dietary habits, but also epigenetic factors and de novo mutations, seem to play a more significant role in the etiology of complex diseases than previously assumed.

Second, there are apparently no common genetic risk factors for the vast majority of common diseases—the CD/CV hypothesis is therefore misguided. For intellectual disability and autism, but also for schizophrenia and probably other common conditions, the lack of common associated markers this can be explained by the reduced fecundity of affected individuals (Power et al. 2013). Therefore, under equilibrium conditions, mutations causing these disorders will be rapidly lost from populations and replaced by others, which is why attempts to infer genetic relationships between psychiatric disorders from shared associated markers were bound to fail (Lee et al. 2013).

Moreover, as pointed out cogently and indeed irrefutably, even the concept underlying the HapMap project (Lander 1996; Terwilliger and Weiss 1998)—that is, to search for conserved haplotype blocs in the genome to reduce the number of markers needed for GWASs—turned out to have serious limitations (Weiss and Terwilliger 2000; Terwilliger and Hiekkalinna 2006).

Finally, the complexity of common diseases is often not due to multifactorial inheritance, but to genetic heterogeneity, because many clinically uniform disorders result from defects in different genes, with intellectual disability (ID) being the most heterogeneous example by far. Extrapolation from the number of known X-linked, autosomal dominant and recessive forms of ID suggests that the total number of genes implicated in ID will run into the thousands (Ropers 2010).

Because several of these facts and arguments are not new, one cannot help wondering why they have not been taken into consideration before reorienting human genome research on common disorders, and why it took almost 15 years before the most fervent supporters of this decision admitted the failure of this strategy (Zuk et al. 2012). Already in 2004, the estimated number of protein-coding human genes had dropped to 25,000 or less (International Human Genome Sequencing Consortium 2004), and soon afterward, high-throughput next-generation sequencing techniques had set the stage for the serial elucidation of single-gene disorders.

## RARE DISEASES REENTER THE STAGE

For obvious reasons, X-chromosomal disorders were among the first to be studied, but after the cloning of the fragile X gene in 1991 (Verkerk et al. 1991), several years elapsed before the systematic search for other causes of X-linked ID picked up speed (Ropers and Hamel 2005). A little later, systematic research into recessive ID was triggered by homozygosity mapping in consanguineous families showing that this condition is extremely heterogeneous (Najmabadi et al. 2007; Kuss et al. 2011). The elucidation of dominant forms of ID received a massive boost when next-generation trio sequencing revealed that, in outbred populations, dominant de novo mutations are the predominant cause of ID (Vissers et al. 2010).

During the past decade, numerous large studies were performed to identify molecular defects underlying ID and related disorders (Najmabadi et al. 2011; Alazami et al. 2015; Karaca et al. 2015; Scott et al. 2016; Riazuddin et al. 2017; Harripaul et al. 2018; Hu et al. 2019). For X-linked (Hu et al. 2016; Martin et al. 2021) and autosomal dominant forms of ID, the yield of novel genes has been decreasing (https://www.sanger.ac.uk/collaboration/deciphering-developmental-disorders-ddd/) suggesting that the size of the relevant gene pools is limited and that most of the respective genes have already been identified. In contrast, despite many large studies performed in populations from the so-called “consanguinity belt” (Bittles 2001), the relative yield of novel genes for recessive forms of ID has been remarkably stable over time, and the overlap between subsequent samples from the same population or between unrelated ones is still small (Harripaul et al. 2018; Hu et al. 2019). In line with theoretical considerations (Ropers 2007), these observations indicate that most Mendelian disorders are recessive, and that their molecular causes are still largely unknown.

According to recent estimates, based on counts involving the entire human genome except the gene-poor Y chromosome (Nurk et al. 2021), there are almost 20,000 protein-coding genes in the human genome—that is, fivefold less than estimated at the start of the HGP. Because almost all are shared with other mammals and even vertebrates, it is very likely that at least “certain mutations in every non-redundant gene will have [negative] phenotypic consequences, either constitutively or in response to specific environmental challenges” (Chong et al. 2015). In other words, nearly all of these should be (at least conditional) disease genes. Because so far, less than 5000 human genes with disease-causing mutations have been identified, this suggests that there are at least twice as many waiting to be detected. Given that in the United Kingdom whole-genome sequencing (WGS) has been adopted as routine diagnostic measure by the NHS (Turnbull et al. 2018) and other countries are about to follow suit, it is a safe bet that henceforth the bulk of relevant genetic and clinical data needed to identify novel genes and their functions will be generated through health care and that the time for large gene hunting expeditions is over.

At present, even long-read WGS techniques have not revealed causative molecular defects in more than, say, two-thirds of diagnostic cases. But because of the unexpected frequency and complexity of large genome rearrangements revealed by optical mapping (Mantere et al. 2021) and the significantly reduced error rate and improved throughput of very-long-read nanopore sequencing (Miller et al. 2021), this could change soon.

Still, it is quite possible that a substantial portion of the missing genetic factors will not cause disease on their own but only in concert with others, resulting in di-, tri-, or even more complex polygenic disorders. Since 2018, when so-called polygenic risk scores (PRSs), calculated by integration of odds ratios for hundreds or thousands of disease-associated SNPs, were presented as markers of complex diseases (Khera et al. 2018), it has become clear that in general, their diagnostic and predictive value is much lower than that of mutations causing monogenic disorders. Like GWASs, PRSs are also plagued by stratification, and they are particularly unsuitable as starting points for unraveling the pathogenesis of complex diseases. For the vast majority of the variants that are associated with complex disease, the risk conferred by individual markers is small, and they are difficult to identify—two compelling reasons for not losing interest in the elucidation of single-gene disorders.

## WHAT THIS ISSUE CONTAINS—AND WHAT NOT YET

Celebrating this year's Rare Disease Day, this Special Issue of *CSH Molecular Case Studies* comprises four invited Commentaries or Perspectives on important developments in this field as well as more than a dozen short publications dealing with rare diseases.

Rachel Alvarez's “Patient perspective: my rare disease journey” sets the stage with a deeply moving account of her own disease history, her indefatigable fight for a correct diagnosis, and her determination to stay alive and to make the best of it for herself and others. As the Executive Director of Cure CMD, an international registry pushing research and therapy of congenital muscle disease, she reminds us that many “rare disorders” are severe, difficult to diagnose clinically, and often without a known molecular cause, and that despite recent progress in disease management (see van Karnebeek et al., this issue), there is presently no cure for most of these disorders. Therefore she emphasizes the importance of defining standards of care for rare diseases and their manifestations.

Although whole-exome sequencing (WES) and, more recently, WGS have added a new dimension to the diagnosis and elucidation of rare diseases, on their own these methods do not reliably detect mutations interfering with normal splicing nor regulatory mutations altering gene expression. In their Perspective entitled “Toward transcriptomics as a primary tool for rare disease investigation,” Stephen Montgomery et al. review the recent literature and the impacts of sequencing RNA in a diagnostic context, concluding that “interrogation of multiple cell types through transcriptomic profiling may be an effective approach to enhancing diagnostic yield in rare disease.”

In the Perspective entitled “Personalized medicine for rare genetic disorders: can we make it happen?,” van Eeghen et al. provide an overview of personalized medicine for rare diseases, with a focus on inherited disorders of metabolism (IDMs). Because numerous IDMs cause neurodevelopmental disorders, they argue that recent progress in this field will also improve the chances for prevention, early detection, and treatment of early-onset brain dysfunction. Three separate articles in this issue (Coughlin et al.; Hauth et al.; Nguyen et al.) deal with monogenic disorders of metabolism that lead to neurodevelopment disorders, thereby supporting the validity of this concept.

Our present inability to reliably identify pathogenic variants is one of the central problems hampering the elucidation of rare diseases and genetic diagnosis. Approaching the problem from the other end, databases listing variants from exomes and genomes from very large cohorts of apparently healthy individuals have been generated to identify, or at least enrich, nondamaging variants (see Genome Aggregation Database, https://gnomad.broadinstitute.org; or the TOPMed Bravo database, https://bravo.sph.umich.edu/). Variants that are conserved between humans and other primates have been exposed to a far more stringent purifying selection because of the evolutionary distance separating these species; therefore, such shared SNVs (Sundaram et al. 2018), but also conserved structural variants, are likely benign (Kleinert and Kircher 2021).

In spite of the numerous published algorithms to predict the pathogenicity of genetic variants (e.g., see Niroula and Vihinen 2019) and the ongoing clinical implementation of WGS, not even 100 thousand of the nearly 10 billion theoretically possible SNVs in the human genome have been listed to date as pathogenic or probably pathogenic in the ClinVar database (Landrum et al. 2018), the most important diagnostic reference used by geneticists world-wide. Since the development of CRISPR–Cas9-mediated saturation genome editing, the functional consequences of all possible variants in genes or given genomic segments can also be tested experimentally (Findlay et al. 2014, 2018), and although it is not a panacea, this potent approach is rapidly gaining popularity (for review, see Findlay 2021). Finally, thanks to the recent success of artificial intelligence-based algorithms to reliably deduce the three-dimensional structure of proteins from their amino acid (and, hence, DNA) sequence (e.g., see Jumper et al. 2021), there is reason to believe that this breakthrough will also further our ability to distinguish pathogenic from neutral variants and will prioritize them for functional studies. Given the importance and complexity of this theme, which merits a more extensive presentation, it will be the topic of a separate review, to be published in the next issue of this journal.

In view of these promising developments Stephen Kingsmore has appropriately called his Perspective “2022: a pivotal year for diagnosis and treatment of rare genetic diseases.” Apart from addressing the highly successful introduction of ultra-rapid WGS for critically ill neonates and infants and decreasing sequencing costs, his central themes are the equitable implementation of WGS, realized only in the United Kingdom so far, but soon in Germany, too, and the urgent need to educate medical personnel for the age of Genomic Medicine that has already begun.

All case studies presented in this issue are related to rare diseases. For each of these, there is a special reason why it has been included, often because it is educative in one way or the other, such as the detection of a recessive founder mutation that appears to be specific to patients with Syrian ancestry (Hassan et al.), the variable intrafamilial expressivity of a heterozygous *TAOK1* frameshift variant (Hunter et al.), ethylmalonic encephalopathy masquerading as meningococcemia (Horton et al.), the unexpected detection of a somatic *FGFR1* mutation in cultured fibroblasts of a patient with closed spinal dysraphism (Kautto et al.), or just the description of a patient with a novel, not previously reported mutation. *CSH Molecular Case Studies* is one of the few journals accepting plausible associations between novel DNA variants and well-described disease phenotypes, even if only supported by a single case. The reason is that is takes such first observations to trigger the search for matching cases, as amply documented by our years of contact with readers of a pioneering paper reporting all sequence variants identified by next-generation sequencing in a large cohort of families with autosomal recessive ID (Najmabadi et al. 2011).

## CONCLUSIONS AND OUTLOOK

“Tackling rare diseases through research and to enable all people living with a rare disease to receive an accurate diagnosis, care, and available therapy within 1 yr of coming to medical attention” was the vision of the International Rare Diseases Research Consortium (IRDiRC), a global collaborative initiative launched in 2011 by the European Commission and the U.S. National Institutes of Health (https://irdirc.org/who-we-are/). Today, a decade later, rare diseases are finally getting the attention they always deserved (Antonarakis and Beckmann 2006) but had not received for many years. Even the German government, one of the last in Europe with a blind eye for rare diseases, has recently adopted a law to implement WGS into health care (see Kingsmore, this issue). At long last, it has also joined the European “1+ Million Genomes” project (https://digital-strategy.ec.europa.eu/en/policies/1-million-genomes) realizing that for the future of genome medicine and research and their successful interplay, international exchange and data sharing is essential.

Technological innovations and novel algorithms, but also databases for pathogenic or benign variants, have drastically reduced the time to diagnosis, and thanks to affordable and fast very-long-read sequencing, genetic diagnoses based on the sequence of the complete, truly personal genome have appeared on the horizon (Nurk et al. 2021). Although at present, the vision of IRDiRC to reduce the time to diagnosis and treatment to less than 1 year is mostly met for patients and families contacting academic or other specialized centers for rare diseases, some patients arrive there only after long diagnostic odysseys. This illustrates the urgent need to establish Genome Medicine as important aspect of medical education and training (see Kingsmore, in this issue), because the difficult truth is that in many countries, the infrastructure and training for referral to such centers is simply not there. Finally, by far not all individuals undergoing state-of-the-art diagnostics will receive a clear diagnosis, because either the underlying gene defect or the significance of identified variants may still be unknown.

This reminds us that despite the remarkable progress and the growing recognition of rare diseases as key to the understanding of gene and genome function, even its original aims defined a decade ago have not been achieved yet, and not only the identification of hitherto unknown disease genes, but above all the elucidation of their function, remains an enormous challenge.

### Competing Interest Statement

The authors have declared no competing interest.

### Acknowledgments

The authors thank Stephen Kingsmore and Elaine Mardis for their helpful comments on an early version of this editorial and for strategic advice, Thomas Wienker for thoroughly reading and discussing the penultimate version, Ada Hamosh and Joanna Amberger for alerting us to relevant publications, Laureen Connell for numerous discussions concerning the Special Issue, and Gwendolin Ropers for her patience.

## Footnotes

3 Referred to here as “rare diseases,” although collectively they are all but rare.
